# Prospective associations of social self-control with drug use among youth from regular and alternative high schools

**DOI:** 10.1186/1747-597X-2-22

**Published:** 2007-07-14

**Authors:** Pallav Pokhrel, Steve Sussman, Louise Ann Rohrbach, Ping Sun

**Affiliations:** 1 Department of Preventive Medicine, Institute for Health Promotion and Disease Prevention Research, University of Southern California, Los Angeles, UK

## Abstract

**Background:**

This study examined the one year prospective associations between adolescent social self-control and drug outcomes (cigarette use, alcohol use, marijuana use, hard drug use, and problem drug use) among adolescents from regular and continuation high schools. In our previous cross-sectional study, poor social self-control was found to be associated with higher drug use, controlling for 12 personality disorder categories. In this study, we attempted to find out (a) whether lack of social self-control predicted drug use one year later, and (b) whether drug use at baseline predicted social self-control one year later.

**Methods:**

We surveyed 2081 older adolescents from 9 regular (N = 1529) and 9 continuation (alternative) (N = 552) high schools in the Los Angeles area. Data were collected at two time points in an interval of approximately 1 year.

**Results:**

Past 30-day cigarette smoking, marijuana use, hard drug use, and problem drug use at baseline were found to predict lower social self-control at follow-up, controlling for baseline social self-control and demographic variables. The effect of problem drug use as a one-year predictor of social self-control was found to be moderated by school type (regular or continuation high school), such that the relationship was significant for continuation high school students only. Conversely, social self-control was found to predict past 30-day alcohol use, marijuana use, and problem drug use, controlling for baseline drug use and demographic variables. For alcohol use, marijuana use, and problem drug use outcomes, school type was not found to moderate the effects of social self-control, though an interaction effect was found regarding cigarette smoking. Social self-control was a significant predictor of cigarette use only at regular high school.

**Conclusion:**

The results indicate that social self-control and drug use share a reciprocal relationship. Lack of social self-control in adolescents seems to result in increased drug use, which in turn is likely to further decrease social self-control. Thus, it seems that social self-control is an alterable cognitive-behavioral attribute which can be improved through skill-based interventions in order to prevent drug use among adolescents. Policies aimed at preventing drug abuse among adolescents may benefit from institutionalizing social self-control skills training.

## Background

Lack of self-control is importantly related to drug involvement [e.g., [1,2]]. Lack of self-control among teens is a strong predictor of heavy drinking, tobacco use, other substance use, as well as perpetration of personal and property crimes [3]. Generally, lack of self-control refers to one's tendency to act without thinking [4] or act on immediate small rewards in preference over delayed large rewards [5,6]. In our previous cross-sectional study [3], a 10-item self-report measure of "social self-control" was examined for its association with substance use, controlling for its associations with 12 personality disorder indices and 4 demographic variables (White ethnicity, Latin ethnicity, socioeconomic status, and male gender) among a sample of 1050 continuation (alternative) high school youth. Social self-control was found to be associated with 30-day cigarette smoking, alcohol use, marijuana use, and hard drug use, controlling for these other variables. The most consistent concurrent predictors of substance use were male gender, antisocial personality disorder, and social self-control. These results highlighted the importance of social self-control as a unique concurrent predictor of drug use among continuation high school students. The present study provides a prospective extension of the previous study and also examines whether drug use variables at baseline predict social self-control one year later. In addition, the present study examines whether relationships between social self-control and drug use variables are similar for both Regular High School (RHS) and Continuation High School (CHS) students. CHS in California is attended by youth that are not able to remain in mainstream education for functional reasons (e.g., truancy, lack of credits, drug use; [7]).

It appears that self-control is a multidimensional construct [8]. Studies in the field have used a variety of self-report measures [e.g., [2,9-11]]. For example, Wills et al. [11] measured self-control in terms of behavioral self-control (e.g., problem-solving, impulsiveness) and emotional self-control (e.g., "soothability," sadness control), using the 20-item Kendall-Wilcox inventory [9] among a number of other scales. In addition, researchers have often divided self-control into good (e.g., planning) and poor (e.g., impulsivity) self-control [e.g., [12,13]]. Social self-control [3] is a unique measure of self-control that measures self-control specifically in relation to interpersonal interactions. Although a few recent studies have attempted to examine the relationship between good and poor self-control and adolescent drug use over a period of time [e.g., [13,13]], to our knowledge, no study so far seems to have examined the predictive relationship between social self-control and drug use.

We propose two hypotheses in the present study. First, we hypothesize that youths low in social self-control would be more likely to use drugs one year later, controlling for baseline drug use and demographic variables, and regardless of type of school. Our assumption is that youth who tend to alienate others through lack of social self-control skills may differentially associate with peers who are more tolerant of their deviant behavior (e.g., drug users). Second, we hypothesize that baseline drug use will fail to predict social self-control one year later, controlling for baseline social self-control and demographic variables. Empirical evidence in the literature suggesting a predictive relation between drug use and low self-control among adolescents does not seem compelling. Gottfredson & Hirschi's [1] consider individuals' social self-control to be relatively stable across time. According to them [1], under similar circumstances, compared to individuals with high self-control, individuals with low self-control are more likely to commit crime and other analogous behaviors (e.g., substance use) all through life. They propose that quality of social environment (i.e., family environment) children are exposed to when they are young determines their level of self-control [1], which is likely to remain stable across developmental stages. Studies have found empirical support for Gottfredson & Hirschi's [1] claim that an individual's self-control does not change with his or her age or differing contexts of residence [e.g., [8]]. Thus, if self-control is similar to a personality trait, then drug use or any other environmental influence (e.g., deviant peer association) should not alter social self-control over time.

Examining the relationships between social self-control and drug use among adolescents has important policy implications. Social self-control as a prospective predictor of drug use among adolescents would suggest that policies concerning adolescent drug use prevention need to consider social self-control as a variable of importance. Furthermore, finding out that drug use is not predictive of social self-control at a later time would support, to some extent, the notion that self-control is more or less stable among adolescents. However, finding out that drug use is predictive of social self-control would suggest that the variable is likely to alter according to the individual's environment and lifestyle. Either finding has policy implications in that, if social self-control is taken to be a personality attribute, then drug use prevention programs targeting self-control may need to target young children and stress family-based interventions, whereas if social self-control is taken to be a variable amenable to change at the adolescent stage of development, then prevention programs may need to consider institutionalizing school-based programs that impart social self-control skills.

## Methods

### Subjects and data collection

Nine school districts from two counties in southern California were recruited for participation in this study. In order to be eligible to participate, districts needed to contain at least one RHS and one CHS, each with an enrollment between 50 and 2000 students. The CHS and RHS were selected from the same school districts (one RHS and one CHS from each district) as 9 pairs of schools.

All students in the randomly selected classrooms first completed project participation information cards that contained their name, address and phone number. Then, students were provided with parental consent forms to take home for their parents' signature, indicating approval or refusal of student participation in the study surveys. Subjects also assented to their involvement. The homes of those students for whom no consent form was returned were called by project staff to request verbal parental consent for survey participation.

A total of 3908 high school students were enrolled in the selected classrooms. Of these, 2751 (70.4% of the enrollment roster) completed pretest questionnaires (1902 RHS and 849 CHS subjects). There were several reasons why we did not have access and, therefore, could not collect data from all enrolled students, including chronic absenteeism (i.e., participant information cards could not be filled out after three weeks of daily attempts; approximately 80% of those not enrolled in the study), either the parent or the student declined participation (approximately 5% of those not enrolled in the study), and students were absent on testing days (which involved a full week of daily attempts at reaching students for pretest and posttest, respectively; approximately 15% of those not enrolled in the study). In general populations, those absent on testing day report more problem prone characteristics [14]; however, among populations at higher risk, those not surveyed at posttest fail to differ greatly from the full sample [15]. No incentive was provided for participation.

Of the students who completed pretest questionnaires, 2081 also completed the follow-up questionnaire an average of 16.5 months later. The sample of 2081 students (N_RHS _= 1529; N_CHS _= 552) constitutes our analysis sample. As with previous studies, our follow-up sample for continuation high school was 65.0% of students assessed at baseline [7]. Subject retention at follow-up was higher for the RHS sample: 80.4% of the baseline RHS sample. The follow-up surveys were conducted by telephone using an interview format. The interviewers (previously unknown to the subjects) contacted the subjects at home, read the questionnaire, and recorded the responses in a survey form. Survey forms and response categories were identical to the in-school questionnaire format used at baseline, and test not to show differences in response patterns [see discussion in [16]].

### Measures

Demographics were assessed using an ethnic indicator (e.g., "what is your ethnic background?" Followed by 6 response options, including an open-ended "Other" option), gender, and parental education indicators (the highest educational level reached across father/step-father or mother/step-mother was measured using 6-point scale, ranging from not completed elementary school to completed graduate school).

Ten social self-control items were used, as described in our previous study [3]. The items were set on a 4-point scale from "never" to "always," and tap behavior in which one seems driven to social excitement even though it distances oneself from social harmony, involving open expression of whatever it is that one feels at the moment which is likely to alienate others, and a desire for the social world to adjust to one's behavior. Example items include: "I enjoy arguing with people," "If I think something someone says is stupid I tell them so," and "My mouth gets me in trouble a lot" (alpha = .73 for entire sample at baseline; alpha = .74, for RHS; alpha = .68, for CHS).

Substance use was measured using single fill-in-the blank item measures. Participants were asked "how many times have you used cigarettes in the last 30 days?" Thirty-day use was also asked in regards to alcohol, marijuana, and 6 categories of hard drugs (which were summed together to form an index: cocaine, stimulants, inhalants, hallucinogens, ecstasy, and other). Subjects were provided with eight response categories, which included 0–10, 11–30, 31–50, 51–60, 61–70, 71–80, 81–90, and 91–100+ times. These drug use behavior items were adapted from previous self-report questionnaires [e.g., [17-20,14]] and have been generally found to have 2-week test-retest reliability of above 0.75 [21]. An index of past 30-day problem drug use was composed of the 11-item Problem Consequences Subscale of the Personal Experience Inventory (PEI; these 4-point items were averaged; [22]). This subscale gives good discriminant validity between interview-derived diagnostic groups (biserial correlation = 0.72; see [21]).

### Attrition analysis

Single sample t-tests and chi-square test for specified proportions were calculated for all variables examined in the study comparing the analysis sample and the full baseline sample for each school type (RHS and CHS). For either school type, no statistically significant differences were detected between the samples on age (p = 0.14, for RHS; p = 0.14, for CHS), gender (p = 0.13, for RHS; p = 0.14, for CHS), ethnicity (p = 0.63, for RHS; p = 0.69, for CHS), parental education (p = 0.70, for RHS; p = 0.76, for CHS), cigarette use (p = 0.25, for RHS; p = 0.42, for CHS), alcohol use (p = 0.38, for RHS; p = 0.96, for CHS), marijuana use (p = 0.28, for RHS; p = 0.54, for CHS), hard drug use (p = 0.15 for RHS; p = 0.63, for CHS), problem drug use (p = 0.87, for RHS; p = 0.71, for CHS), and social self-control (p = 0.78, for RHS; p = 0.66, for CHS). These results imply that attrition bias did not pose serious threats to the present study.

### Analyses and results

Table 1 and Table 2 show the demographic and drug use characteristics of the analysis sample at baseline and follow-up for RHS and CHS students. The Pearson correlation coefficients among cigarette, alcohol, marijuana, and hard drug use for RHS subjects at baseline ranged from 0.25 (between alcohol and hard drug use; p < 0.0001) to 0.41 (between marijuana and cigarette use; p < 0.0001); for CHS subjects at baseline correlations ranged from 0.29 (between alcohol and hard drug use; p < 0.0001) to 0.46 (between alcohol and marijuana use; p < 0.0001). At follow-up, the correlations among the drugs ranged from 0.28 (between alcohol and hard drug use; p < 0.0001) to 0.49 (between hard drug and marijuana use; p < 0.0001) for RHS subjects and from 0.25 (between alcohol and hard drug use; p < 0.0001) to 0.39 (between marijuana and cigarette use; p < 0.0001). For both RHS and CHS samples, a statistically significant increase in social self-control was found to have taken place between baseline and follow-up (p < 0.0001).

**Table 1:** Demographic, baseline drug use, and social self-control characteristics of the analysis sample.

		RHS (N = 1529)	CHS (N = 552)
Mean age***		14.8 (SD = 0.86)	16.8 (SD = 0.74)
Gender***			
	% Male	51.6	57.2
	% Female	48.4	42.8
Ethnicity***			
	% Latino	60.6	71.2
	% Non-Latino	39.4	28.8
Parents education***			
	% Some college or below	69.7	80.0
	% Full college or above	30.3	20.0
% using cigarette***		8.3	35.4
% using alcohol ***		31.3	58.0
% using marijuana***		13.7	41.0
% using hard drug***		4.90	17.6
Mean Problem drug use***		1.09 (SD = 0.24)	1.21 (SD = 0.33)
Mean social self-control		2.87 (SD = 0.50)	2.88 (SD = 0.51)

Note: *p < 0.05; **p < 0.001; ***p < 0.0001; SD = Standard deviation; % using drug pertains to any use in last 30 days; RHS = Regular High School; CHS = Continuation High School

**Table 2:** Drug use and social self-control characteristics of the analysis sample at one year follow-up.

	RHS (N = 1529)	CHS (N = 552)
% using cigarette***	11.8	39.0
% using alcohol***	34.0	58.1
% using marijuana***	12.9	31.5
% using hard drug***	5.59	12.6
Mean Problem drug use***	1.06 (SD = 0.20)	1.14 (SD = 0.30)
Mean social self-control***	3.08 (SD = 0.46)	3.07 (SD = 0.45)

Note: *p < 0.05, **p < 0.001, ***p < 0.0001; SD = Standard deviation; % using drug pertains to any use in last 30 days; RHS = Regular High School; CHS = Continuation High School.

Ten models were examined using PROC MIXED and PROC GLIMMIX on SAS statistical software (Version 9.1). PROC MIXED and PROC GLIMMIX analytical techniques account for random model effects in regression analyses with continuous and dichotomous outcome variables respectively. Accounting for random model effects was important to our analyses as our subjects were nested within schools. The first five models examined the prediction of drug use one year later (cigarette smoking, alcohol use, marijuana use, hard drug use, or problem drug use) from baseline social self-control, controlling for baseline drug use, age, gender, Latino ethnicity, school type (RHS or CHS), and parents' education. In addition, each model also tested for the interaction effect of school type on the relationship between social self-control and specific drug use by including an interaction term in the model. All variables were centered on their means and specified a standard deviation of 1. The interaction term was created after standardizing the variables [23]. Since the drug use variables were highly skewed towards non-use, they were dichotomized into having used a specific drug in the past 30 days or not.

The results based on the first five analytical models are shown in Table 3. Social self-control predicted alcohol use, marijuana use, and problem drug use controlling for baseline drug use and the five demographic variables. Although not statistically significant, social self-control at baseline was inversely associated with both cigarette use and hard drug use at follow-up. No significant interaction was detected between social self-control and school type for alcohol use (p = 0.10), marijuana use (p = 0.21), hard drug use (p = 0.44), and problem drug use (p = 0.39). However, the interaction between school type and social self-control was found to be statistically significant for cigarette use (p = 0.01). When examined separately by school type, social self-control at baseline was found to predict cigarette use significantly at follow-up only among RHS subjects (OR = 0.79; 95% CI = 0.67-0.92).

**Table 3:** Social self-control at baseline as a predictor of drug use one year later, after controlling for age, female gender, Latino ethnicity, parents' education, school type, and baseline drug use.

	Odds Ratio (95% Confidence Interval)				Standardized *β*
	Cigarette smoking	Alcohol use	Marijuana use	Hard drug use	Problem drug use

Baseline drug variable	2.13*** (1.94–2.35)	1.82*** (1.65–2.01)	1.92*** (1.74–2.10)	2.05*** (1.87–2.25)	0.35***
Social self-control	0.91^a ^(0.79–1.04)	0.80*** (0.72–0.89)	0.81*** (0.71–0.91)	0.89 (0.76–1.05)	-0.09**
Age	1.01 (0.89–1.14)	0.99 (0.90–1.09)	0.92 (0.82–1.04)	0.74** (0.62–0.88)	-0.07**
Female gender	0.86** (0.76–0.96)	0.83** (0.76–0.92)	0.80** (0.71–0.90)	0.94 (0.82–1.07)	-0.06*
Latino ethnicity	1.05 (0.91–1.20)	1.09 (0.99–1.20)	1.02 (0.91–1.15)	1.13 (0.98–1.33)	0.02
Parents' education	1.04 (0.93–1.17)	0.93 (0.85–1.02)	1.00 (0.89–1.12)	1.04 (0.91–1.20)	-0.02
School type (RHS = 0; CHS = 1)	7.69^b^*** (3.87–15.27)	2.94*** (2.67–3.24)	3.86*** (2.55–5.94)	4.01*** (1.45–11.12)	0.35**

Note: *p ≤ 0.05; **p ≤ 0.001; ***p < 0.0001; All independent variables were centered on their means.

^a ^Interaction between school type and social self-control statistically significant (p = 0.01). The reported OR is when school type = 0 (school type was centered on its mean).

^b ^Interaction between school type and social self-control statistically significant (p = 0.01). The reported OR is when social self-control = 0 (social self-control was centered on its mean).

The second five models examined the prediction of social self-control at one year follow-up from the level of drug use (cigarette smoking, alcohol use, marijuana use, hard drug use, or problem drug use) at baseline, controlling for baseline social self-control, school type, female gender, Latino ethnicity, and parents' education. All variables were standardized prior to running the models. The term for interaction between specific drug variable and school type was also included in each model. The results of this set of analyses are summarized in Table 4. The inverse association between drug use at baseline and social self-control at follow-up was found to be significant for cigarette use (p = 0.05), marijuana use (p = 0.04), hard drug use (p = 0.04), and problem drug use (p = 0.002). No interaction was found taking place between school type and cigarette use (p = 0.26), alcohol use (p = 0.38), marijuana use (p = 0.84), and hard drug use (p = 0.48). However, school type was found to modify the effect of problem drug use at baseline on social self control at follow-up (p = 0.03). Further analysis showed that higher problem drug use at baseline was significantly associated with lower social self-control at follow-up for CHS students (standardized β = -0.13; p < 0.001) but not for RHS students (β = -0.03; p = 0.23).

**Table 4:** Effects of baseline drug use on social self-control one year later, after controlling for baseline social self-control, age, female gender, Latino ethnicity, parents' education, and school type.

	Standardized *β*				
	Cigarette smoking	Alcohol use	Marijuana use	Hard drug use	Problem drug use

Baseline social self-control	0.50***	0.50***	0.50***	0.50***	0.49***
Drug use	-0.04*	-0.03	-0.04*	-0.04*	-0.07^a^*
Age	0.06*	0.06*	0.06*	0.06*	0.05*
Female gender	-0.04*	-0.03	-0.04*	-0.03	-0.04*
Latino ethnicity	-0.001	-0.006	-0.007	-0.005	-0.004
Parents' education	0.005	0.004	0.004	0.007	0.002
School type	0.01	0.008	0.007	0.007	0.0008

Note: *p ≤ 0.05; ***p < 0.0001; All independent variables were centered on their means;

^a ^Interaction between school type and problem drug use was found to be statistically significant (p < 0.03). The reported *β *is when school type = 0 (school type was centered on its mean).

## Discussion

Higher levels of social self-control may be protective against drug use experimentation [e.g., [24,7]]. In Sussman et al. [3], which was a cross-sectional study, high social self-control was inversely related to drug use, controlling for relatively unchangeable disorders of personality. This result suggested to us that social self-control is not merely a facet of a problem personality. The present study attempted to examine whether the findings of the previous study would replicate longitudinally in a larger, more varied sample. Extending the sample from the previous study which included only the "high risk youth" (i.e. continuation high school students), the present study included both regular and continuation high school students. In addition, the present study also examined whether drug use had prospective effects on social self-control.

Contrary to our assumption that social self-control might be relatively stable over time, our findings indicated that the mean social self-control for both RHS and CHS samples increased significantly from baseline to follow-up. This increase in social self-control suggests that rather than being an immutable personality characteristic, social self-control is likely to be a complex cognitive-behavioral attribute that might improve as one gets older. Wills and colleagues [25,26] have argued that self-control requires a sophisticated level of cognitive and social development which takes place through adolescence.

Our hypothesis that drug use at baseline would fail to predict social self-control one year later was supported only in case of alcohol use. In cases of cigarette smoking, marijuana use, and hard drug use, our results did not support the hypothesis. After controlling for baseline social self-control and demographic variables, cigarette smoking, marijuana use, and hard drug use were found to be inversely related to social self-control at follow-up one year later across both school types. Research in neuropharmacology suggests that persistent use of drugs, including nicotine, may affect the neurophysiology associated with behavioral inhibition through neuroplastic changes [27]. Drug use behaviors are also likely to affect social self-control through differential deviant peer selection and subsequent imitation of socially inappropriate behavior [28]. It is possible that alcohol use failed to predict social self-control because most of the alcohol users may not be abusing the drug. Currently, the extent of research examining drug use as a prospective predictor of self-control seems to be limited. Future research on adolescent self-control and drug use behavior may need to examine the relationship more closely in order to explain the mediators linking drug use to social self-control. As regards the finding that problem drug use at baseline had significant effects on the social self-control of CHS subjects, but not RHS subjects, the implication might be that negative interpersonal consequences of drug use (e.g., trouble with teachers, peers, and parents due to drug use) may further alienate the high risk youth socially and encourage them to behave more irresponsibly in social situations later on.

Our hypothesis that social self-control at baseline would predict drug use a year later was partly supported. Social self-control at baseline showed significant inverse associations with alcohol, marijuana use, and problem drug use one year later, controlling for the specific baseline drug variable and demographic covariates. The effects of baseline social self-control on these drug outcomes were found to be similar across RHS and CHS. However, school type was found to moderate the effects of social self-control on cigarette use. That is, social self-control had a significant inverse effect on follow-up cigarette use for RHS students only. Our failure to detect significant association between social self-control and hard drug use might be due to relatively lower prevalence of hard drug use among our subjects. The results were in the expected direction in the present investigation.

Failure to find a longitudinal association between social self-control and cigarette use among CHS youth could be interpreted as an implication that cigarette use in the CHS context is considered less deviant (or more socially acceptable) than in the RHS context. If it is possible that lack of social self-control predisposes one to alienate others and use drugs as a shared activity that others who also lack social self-control may tend to engage in [29,3], then perhaps the students lacking in social self-control in the CHS social environment do not gravitate toward deviant peers whose deviancy is attributed exclusively to smoking.

To see whether the association between social self-control and cigarette use differed by school type cross-sectionally at baseline, we conducted a regression analysis following the same procedures outlined above (see Results & Analyses) for the longitudinal analyses. No significant interaction was detected between school type and social self-control (p = 0.47). In addition, social self-control was inversely and significantly associated with cigarette use among both RHS and CHS subjects, controlling for demographic covariates (p < 0.001) when the analysis was run separately by school type. This cross-sectional finding was similar to the findings of our previous study. Thus, only further longitudinal studies may be able to elucidate whether lack of social self-control skills is causally related to cigarette use, or that cigarette smoking might reflect contextual differences in appropriateness.

There are several limitations of this study including lack of direct overt behavioral measures and lack of multiple psychometric evaluations of the social self-control measure (e.g., test-retest reliability). Although we tested all the relevant variables for indications of a bias due to attrition, we didn't test several others that were not measured in the study. For example, indicators of SES other than parental education, such as parental income and number of persons living in a house were not measured in the study. In addition, our data may not generalize to all Los Angeles area RHS and CHS. Although students were randomly selected at the classroom level, some selection bias might have been introduced to the data at the level of school, which was based on convenience sampling. Furthermore, about 30% of the students enrolled in the selected classes did not fill out the baseline survey. The fact that these students could have been more likely to show problem behavior [14] might have also biased our data.

One may speculate that training skills like social self-control may be useful in order to prevent the misuse of various prescription drugs, which are often prescribed to adolescents in order to temper disorders and problem behavior [30]. Certainly, study of the relations of these drugs with social self-control would make an interesting topic of future study, and may have some policy implications regarding the costs and benefits of misusing such prescription drugs (e.g., such as methylphenidate, or amphetamine). What is clear from our findings is that youth who learn *not *to alienate others frequently in social interactions may be less likely to revert to drug use.

The body of evidence linking personality and pathological forms of behavioral undercontrol in adolescents to substance use has been growing [e.g., [31-33]]. Although a measure of behavioral undercontrol, social self-control is neither a personality trait as is novelty seeking nor a form of psychopathology. Sussman et al. [3] found that antisocial personality disorder was non-redundant with the social self control measure. However, despite some fundamental differences, the measure of social self-control does share some similarities with DSM-IV diagnostic criteria for Oppositional Defiant Disorder (ODD) [34]. ODD primarily concerns a youth's or a child's behavioral disinhibition as reflected through his or her interaction with adults, whereas social self-control concerns his or her interaction with both peers and adults.

More importantly, compared to the social self-control measure the ODD measure focuses more on the defiant and vindictive aspects of personality (e.g., "Often blames others for his or her mistakes or misbehavior", "Is often spiteful or vindictive"). In total, six social self-control items do not overlap with any of the ODD items. Examples of these include, "I say things that I regret later," "I express all my feelings," and "My mouth gets me in trouble a lot." However, both measures tend to overlap on four items (that is, after overlooking the focus on child-adult relationship in ODD measure) concerning argumentative behavior, anger undercontrol, willful annoying behavior, and touchiness. King et al. [32] found ODD at age 11 to significantly predict the onset of use, regular use, and advanced use experience at age 14 for cigarette, alcohol, and marijuana. Since social self-control overlaps with certain indicators of ODD and does not focus on the diagnosis of psychopathology, social self-control can perhaps be viewed as an indicator of a more common form of behavioral undercontrol. Currently, it is not known whether lack of social self-control may over time develop into a psychopathogical condition in certain types of personality. However, given the fact that social self-control shares similarities with ODD and that it is associated with future substance use, it seems worthwhile to address the problem of behavioral disinhibition at the level of social self-control, especially among younger teens.

## Conclusion

Our study suggests that there might be a reciprocal relationship between drug use and social self-control such that each is likely to affect the other. Low social self-control is likely to result in higher drug use, which in turn is likely to result in lower self-control. Social self-control seems to be alterable and likely to be influenced by adolescents' lifestyle and social environment. Thus, providing adolescents with social self-control skills that utilize cognitive-behavioral techniques to perform social self-control is likely to prevent drug use for a longer term. Project Toward No Drug Abuse (Project-TND), which is a teen drug abuse prevention program uses a social self-control skill training (e.g., teaching impulse control techniques) as a core program component and has proven to reduce drug use significantly among teens of ages 14–19 years and diverse ethnicity [see [35]]. Two of the sixteen adolescent drug abuse prevention principles enlisted by National Institute of Drug Abuse (NIDA) advocate that programs for elementary school children should focus on imparting self-control skills and interpersonal skills [36]. The present study suggested that self-control and social/interpersonal skills are not entirely independent entities, but related in the form of social self-control, which needs to be incorporated in prevention programs designed for high school students. Although future studies would help in order to understand social self-control better, present policies are likely to benefit from recognizing the protective effects of social self-control on drug use.

## Competing interests

The author(s) declare that they have no competing interests.

## Authors' contributions

PP did the statistical analyses and wrote the majority of the revised text.

SS wrote the first draft, edited the revised draft, and provided mentorship throughout.

PS provided mentorship on statistical analyses.

LAR contributed to the methods section.

All authors read and approved the final manuscript.
